# SSIPTools: Software and Methodology for Surface Site Interaction Point (SSIP) Approach and Applications

**DOI:** 10.1021/acs.jcim.1c01006

**Published:** 2021-10-29

**Authors:** Mark D. Driver, Mark J. Williamson, Nicola De Mitri, Teodor Nikolov, Christopher A. Hunter

**Affiliations:** Yusuf Hamied Department of Chemistry, University of Cambridge, Lensfield Road, Cambridge CB2 1EW, United Kingdom

## Abstract

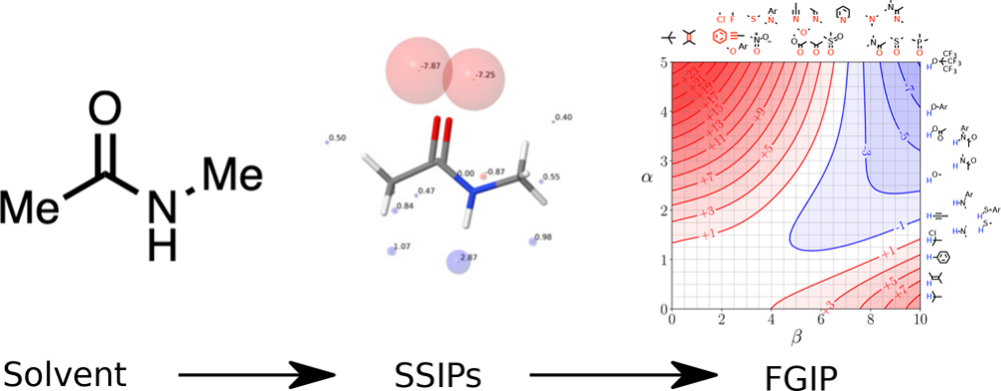

We present the SSIPTools suite of programs. SSIPTools is a collection of software modules enabling the use of the Surface Site Interaction Point (SSIP) molecular descriptors, used for the modeling of noncovalent interactions in neutral organic molecules. It contains an implementation of the workflow for the generation of the SSIP descriptors, as well as the Functional Group Interaction Profiles (FGIPs) and Solvent Similarity Indexes (SSIs) applications, based on the SSIMPLE (Surface Site Interaction model for the Properties of Liquids at Equilibria) approach.

## Introduction

A wide range of condensed phase phenomena are influenced by the formation of noncovalent interactions, which dominate many solvation effects. These interactions govern physical properties such as solubility, miscibility, and vapor pressure,^1−4^ as well as chemical properties such as molecular recognition, supramolecular self-assembly, and rates of chemical reactions.^5−9^ The complexity of the network of coupled equilibria involved in solvation of molecular mixtures in different solvent environments has been a substantial issue in the challenge for the theoretical prediction of solubility. Empirical solvent descriptors have proved valuable in extrapolating experimental data,^5,10−13^ and computational methods have been developed for including solvent effects in *ab initio* simulations of molecular properties.^14−18^

The Surface Site Interaction Point (SSIP) approach was previously developed for understanding the contribution of individual noncovalent interactions in solvation, which is based on experimental studies of pairwise interactions between hydrogen bonded solutes.^19^ The application of the SSIP approach to the calculation of solvent properties uses the Surface Site Interaction for the Properties of Molecules at Equilibrium (SSIMPLE).^20^ With SSIMPLE, the population of free and bound SSIPs can be computed, providing insight into the interactions present at equilibrium, allowing calculation of solvation free energies and prediction of partition coefficients. From the population information, the energy of solute–solute interactions can be computed, as displayed in functional group interaction profiles (FGIPs).^21^ The computed solvation energy of an idealized solute SSIP has also previously been used to develop the Solvent Similarity Index (SSI), a quantitative comparison metric for the assessment of the similarity between two solvent systems.^22^

In this work, we describe the software suite created to automate SSIP description generation^23^ for a molecule. We then describe how this can be applied to the automated calculation, in the SSIMPLE framework, of FGIPs^21^ and SSIs^22^ of neutral organic molecules.

## Footprinting Process: Generating SSIP Descriptions

Within the SSIP approach, a molecule is described by a set of discrete interaction sites. An interaction parameter, ϵ_*i*_, is assigned to each SSIP (referred to as an SSIP value in this work), which is equivalent to the experimentally measured hydrogen bond donor parameter (α) for positive sites or the hydrogen bond acceptor parameter (−β) for negative sites.^19^ The dimensionality of SSIP values is such that ϵ^2^ is a molar energy.

**Figure 1:**
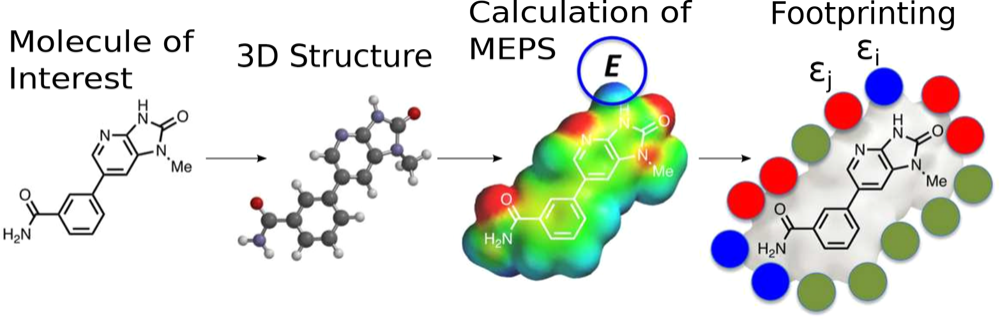
SSIP footprinting. The process starts with a molecule of interest, for which a 3D structure is generated. The MEPS data are then calculated for the molecule, before this is coarse grained to produce a collection of SSIPs which describe the surface interaction sites.

Generation of the SSIP description, as shown in Figure 1, can be decomposed into three discrete units, promoting the development of a modular code base for the workflow. Generation of a 3D structure for the molecule of interest is followed by calculation of molecular electrostatic potential surface (MEPS) of the molecule. Footprinting converts the MEPS data to the SSIP description in the final step (a coarse graining approach described by Calero et al.^23^).

The output of the SSIP footprinting process is an eXtensible Markup Language (XML) file (see SI for details), making the CML format^24^ the appropriate 3D representational format for the structures in this workflow. The molecular electrostatic potential surface (MEPS) data are stored in unformatted cube files.^25^

**Figure 2:**
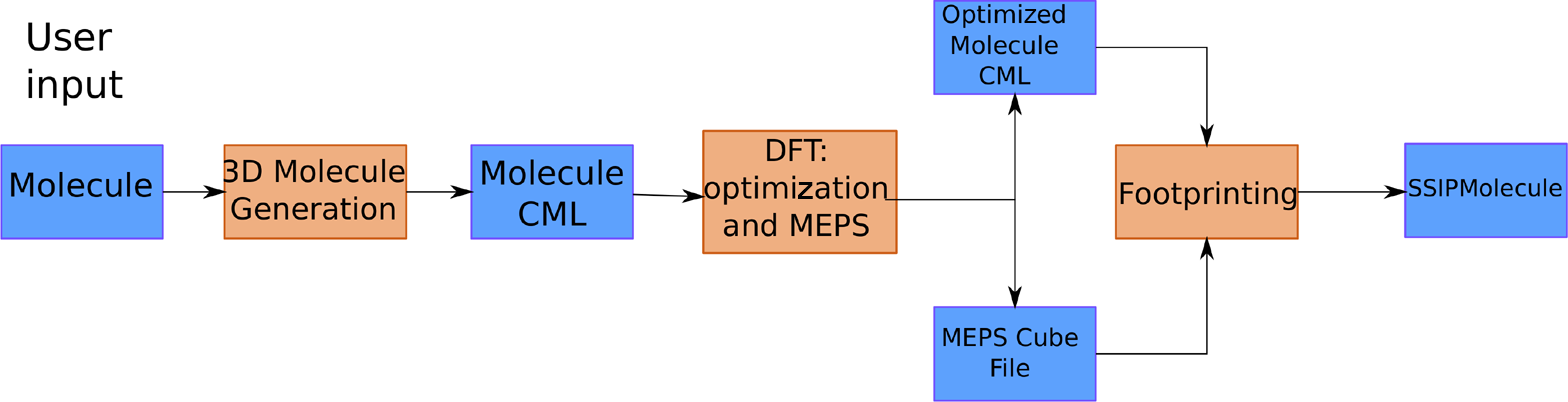
Workflow showing the process of generating a SSIP description for a molecule from input structure to the final XML output. Blue rectangles represent input/output information, and orange rectangles represent computational processes.

Each SSIP is associated with a position on the 0.002 e bohr^–3^ electron density isosurface^23^ of a molecule. Figure 2 shows the modular construction of the computational framework developed to undertake this work. The process uses the aforementioned data format specifications to be used in input/output operations at the interfaces between different computational modules.

The Python library cmlgenerator (see SI for details) created for this work provides the functionality required to generate schema conforming CML input files for the MEPS calculation. Existing 3D structures of target molecules in other input formats (e.g., PDB, mol2, SDF) are converted by cmlgenerator using Open Babel^26^ as a backend. It is also possible to input structures as a 2D SMILES^27^ string representation. To generate a suitable 3D structure, the RDKit^28^ package is used with the ETDKG2^29^ conformer generation and the UFF^30^ force field to select a suitably optimized starting point to be used in the MEPS generation step if no structure is given (a full geometry optimization is carried out in the next step).

Density functional theory (DFT) is used to optimize the molecule geometry and to generate the MEPS on the 0.002 e bohr^–3^ electron density isosurface.^23^ The calculation employs the B3LYP functional^31−34^ and a 6-31G*^35,36^ basis set for all atoms, except bromine, selenium, and iodine, for which 6-311G**^37^ is used, based on work in ref (23). Calculations were run using NWChem^38,39^ and forms the rate limiting step in the workflow. The Python library nwchemcmlutils (see SI for details) was created to provide a simple CLI for the generation of MEPS information (see SI for further details).

**Figure 3:**
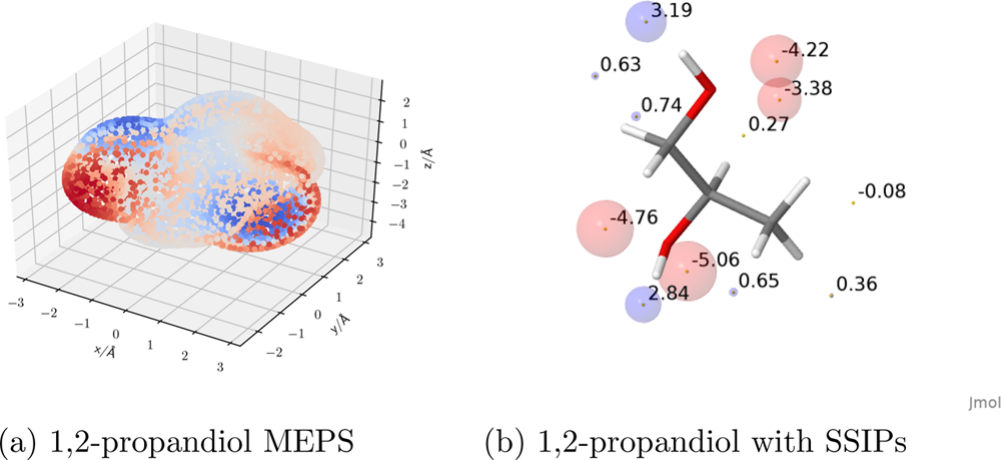
Conversion of the MEPS (left, with no atoms shown) to the SSIPs (right, with atoms shown) for 1,2-propandiol. A color map is used for the MEPS, going from blue (for positive MEPS points) to red (negative electrostatic potentials), with the color intensity representing the magnitude of the MEPS (darker is stronger). (b) Blue is used for positive SSIPs and red for negative SSIPs; the size of each sphere represents its magnitude.

The computed MEPS and 3D structure are then combined during the footprinting process (originally detailed in ref (23)) to produce the SSIP description using the SSIP Java package. Figure 3 contains example output of the footprinting process for 1,2-propandiol. The details of the Java package used for this work, and our previous studies,^21,22^ including algorithmic improvements, are described in the SI.

## Surface Site Interaction Model for Properties of Liquids at Equilbrium (SSIMPLE) and Applications

Interactions between SSIPs are treated in a pairwise manner to describe a liquid or gas phase using SSIMPLE.^20^ The association constant for the interaction of the *i*th and *j*th SSIP, *K*_*ij*_, is given by eq 1.
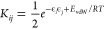
1where *E*_vdW_ = −5.6 kJ mol^–1^.

As we have shown previously,^20,40^ van der Waals interactions between nonpolar molecules are, to a first approximation, a linear function of surface area, so by choosing a description that gives all SSIPs the same area footprint on the van der Waals surface of a molecule a constant value can be used for *E*_vdW_. The interaction energy is made up of a polar term, ϵ_*i*_ϵ_*j*_, and a nonpolar term, *E*_vdW_, which is the energy of the van der Waals interaction between two SSIPs. For repulsive interactions (i.e., ϵ_*i*_ and ϵ_*j*_ have the same sign), it is assumed that a state can be found where the polar sites are misaligned such that only nondirectional van der Waals interactions are made, and the polar interaction term, ϵ_*i*_ϵ_*j*_, is set to zero.

The standard state used to ensure *K*_*ij*_ is dimensionless is the maximum theoretical density of SSIPs, *c*_max_ = 300 M. The value of *c*_max_ is based on the reference volume associated with a SSIP, 5 Å^3^, that was defined using the volume enclosed by the van der Waals surface of a water molecule, which is represented by four SSIPs.^20^ The speciation of all SSIP contacts in the liquid phase can then be calculated.

From this speciation data, the phase transfer energies,^20^ FGIPs,^21^ and SSI^22^ information have previously been derived.

**Figure 4:**
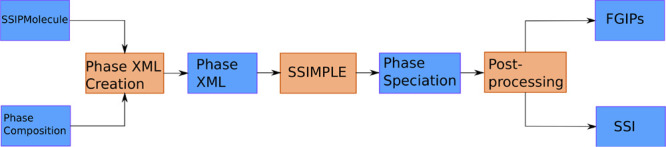
Workflow showing the process of preparing SSIMPLE calculations for applications to FGIP and SSI generation. The phasecalculator interface provides a convenient interface wrapping to automate of the processing involved.

**Figure 5:**
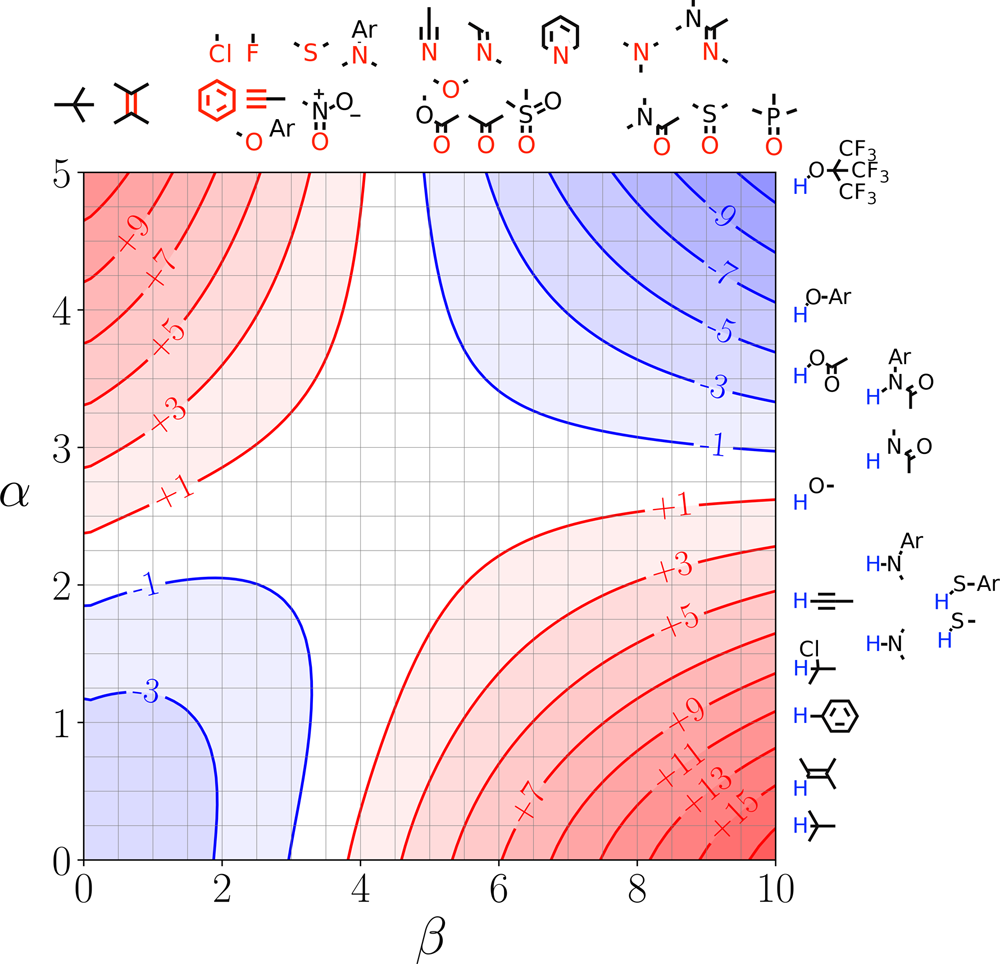
FGIP for the interaction of two solutes in water at 298 K (ΔΔ*G*_FGI_ in kJ mol^–1^). The solute–solute interactions are favorable in the blue region and unfavorable in the red region. Produced using the phasecalculator interface.

The workflow in Figure 4 is entirely encompassed by the phasecalculator module (see SI for details), so that only a description of the solvent mixture (the phase composition) is required as an input when all solvents were previously explored. For instance, only two command line calls of the phasecalculator were required to generate the FGIP for water at 298 K, whose representation is reported in Figure 5, allowing reproduction of the results from.^21^

**Figure 6:**
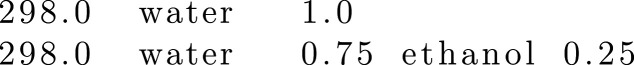
Contents of tsv file required to specify creation of two phases. The first line defines a phase of pure water (χ_water_ = 1.0) at 298 K. This input was used to generate the FGIP in Figure 5. The second line defines a mixed phase of water (χ_water_ = 0.75) and ethanol (χ_ethanol_ = 0.25) at 298 K.

The phase compositions for mixtures to be explored is specified using a tab-separated values (.tsv) file. One phase specification is included per line, with temperature reported in the first column, and each subsequent pair of columns representing a component name and mole fraction. An example of this input file is found in Figure 6, containing two different phase definitions. The file provides the ability to calculate additional properties using the previously defined solvent SSIP descriptions^21^ (see SI for more detail).

## Conclusion

The SSIP approach to molecular descriptions has previously been described along with applications of the SSIMPLE method. The workflow presented here provides a detailed description of the computational tools used to generate the previous work to allow readers to replicate the results and explore novel solvent systems tailored to individual requirements. The publishing of the accompanying software using popular repositories will allow other researchers to use the tools we have developed to explore systems of interest.

## Data and Software Availability

The software described in the paper is hosted at the University of Cambridge gitlab, located at https://gitlab.developers.cam.ac.uk/ch/hunter/ssiptools. All software in the SSIPTools collection is released under an AGPLv3 license for academic use. Enquiries for any nonacademic use of SSIPTools including commercial use should be directed to Cambridge Enterprise: Cambridge Enterprise Ltd., University of Cambridge, Hauser Forum, Three Charles Babbage Rd., Cambridge CB3 0GT, United Kingdom; Tel: + 44 (0)1223 760339; Email: software@enterprise.cam.ac.uk. The Python packages have also been deployed to conda-forge, enabling installation using the anaconda python distribution. This allows installation by the following command: conda install -c conda-forge {package name}. The Python packages in SSIPTools are xmlvalidator, cmlgenerator†, nwchemcmlutils†, ssipfootprint†,phasexmlcreator, phasexmlparser, resultsanalysis, puresolventinformation, solventmapcreator, and phasecalculator†. Packages marked with † are the main packages users will interact with and install (other packages are dependencies that will be installed automatically by anaconda during dependency resolution). The Java SSIP project in SSIPTools has three jar targets: ssip-footprint (ssip when installed as a deb package), which performs the footprinting process described in ref (23), ssip-phasetransfer (phasetransfer when installed as a deb package), which performs the SSIMPLE calculation described in ref (20), and ssip-visualization (ssip-vis when installed as a deb package) which is used for SSIP description visualization. Precompiled artifacts are available for download from the website. It has also been deployed to maven for inclusion in other Java projects.

## Supporting Information Available

Detailed algorithm and extended feature descriptions (PDF)

## References

[ref1] Mannhold R.; Poda G. I.; Ostermann C.; Tetko I. V. Calculation of molecular lipophilicity: State-of-the-art and comparison of log P methods on more than 96,000 compounds. J. Pharm. Sci. 2009, 98, 861–893. 10.1002/jps.21494. 18683876

[ref2] Skyner R. E.; McDonagh J. L.; Groom C. R.; van Mourik T.; Mitchell J. B. O. A review of methods for the calculation of solution free energies and the modelling of systems in solution. Phys. Chem. Chem. Phys. 2015, 17, 6174–6191. 10.1039/C5CP00288E. 25660403

[ref3] Hansen C. M. Hansen Solubility Parameters: A User’s Handbook, 2nd ed.; CRC Press, 2007.

[ref4] Perry R. H. Perry’s Chemical Engineers’ Handbook, 7th ed.; McGraw-Hill, 1997.

[ref5] Taft R. W.; Gurka D.; Joris L.; Schleyer P. R.; Rakshys J. W. Studies of Hydrogen-Bonded Complex Formation with p-Fluorophenol. V. Linear Free Energy Relationships with OH Reference Acids. J. Am. Chem. Soc. 1969, 91, 4801–4808. 10.1021/ja01045a038.

[ref6] Fersht A. R. Enzyme Structure and Mechanism; W.H. Freeman, 1985.

[ref7] Hunter C. A.; Sanders J. K. The Nature of *π-π* Interactions. J. Am. Chem. Soc. 1990, 112, 5525–5534. 10.1021/ja00170a016.

[ref8] Schneider H.-J. Mechanisms of Molecular Recognition: Investigations of Organic Host–Guest Complexes. Angew. Chem., Int. Ed. Engl. 1991, 30, 1417–1436. 10.1002/anie.199114171.

[ref9] Doyle A. G.; Jacobsen E. N. Small-Molecule H-Bond Donors in Asymmetric Catalysis. Chem. Rev. 2007, 107, 5713–5743. 10.1021/cr068373r. 18072808

[ref10] Gurka D.; Taft R. W. Studies of Hydrogen-Bonded Complex Formation with p-Fluorophenol. IV. The Fluorine Nuclear Magnetic Resonance Method. J. Am. Chem. Soc. 1969, 91, 4794–4801. 10.1021/ja01045a037.

[ref11] Abraham M. H. Hydrogen bonding. 31. Construction of a scale of solute effective or summation hydrogen-bond basicity. J. Phys. Org. Chem. 1993, 6, 660–684. 10.1002/poc.610061204.

[ref12] Abraham M. H.; Chadha H. S.; Dixon J. P.; Leo A. J. Hydrogen bonding. 39. The partition of solutes between water and various alcohols. J. Phys. Org. Chem. 1994, 7, 712–716. 10.1002/poc.610071209.

[ref13] Abraham M. H.; Platts J. A. Hydrogen Bond Structural Group Constants. J. Org. Chem. 2001, 66, 3484–3491. 10.1021/jo001765s. 11348133

[ref14] Klamt A. Conductor-like Screening Model for Real Solvents: A New Approach to the Quantitative Calculation of Solvation Phenomena. J. Phys. Chem. 1995, 99, 2224–2235. 10.1021/j100007a062.

[ref15] Klamt A.; Jonas V.; Bürger T.; Lohrenz J. C. W. Refinement and Parametrization of COSMO-RS. J. Phys. Chem. A 1998, 102, 5074–5085. 10.1021/jp980017s.

[ref16] Marenich A. V.; Olson R. M.; Kelly C. P.; Cramer C. J.; Truhlar D. G. Self-consistent reaction field model for aqueous and nonaqueous solutions based on accurate polarized partial charges. J. Chem. Theory Comput. 2007, 3, 2011–2033. 10.1021/ct7001418. 26636198

[ref17] Cramer C. J.; Truhlar D. G. A Universal Approach to Solvation Modeling. Acc. Chem. Res. 2008, 41, 760–768. 10.1021/ar800019z. 18512970

[ref18] Tomasi J.; Mennucci B.; Cammi R. Quantum Mechanical Continuum Solvation Models. Chem. Rev. 2005, 105, 2999–3094. 10.1021/cr9904009. 16092826

[ref19] Hunter C. A. Quantifying intermolecular interactions: Guidelines for the molecular recognition toolbox. Angew. Chem., Int. Ed. 2004, 43, 5310–5324. 10.1002/anie.200301739. 15468180

[ref20] Hunter C. A. A surface site interaction model for the properties of liquids at equilibrium. Chem. Sci. 2013, 4, 1687–1700. 10.1039/c3sc22124e.

[ref21] Driver M. D.; Williamson M. J.; Cook J.; Hunter C. A. Functional group interaction profiles: a general treatment of solvent effects on non-covalent interactions. Chem. Sci. 2020, 11, 4456–4466. 10.1039/D0SC01288B. 34122903 PMC8159447

[ref22] Driver M. D.; Hunter C. A. Solvent similarity index. Phys. Chem. Chem. Phys. 2020, 22, 11967–11975. 10.1039/D0CP01570A. 32412565

[ref23] Calero C. S.; Farwer J.; Gardiner E. J.; Hunter C. A.; Mackey M.; Scuderi S.; Thompson S.; Vinter J. G. Footprinting molecular electrostatic potential surfaces for calculation of solvation energies. Phys. Chem. Chem. Phys. 2013, 15, 18262–73. 10.1039/c3cp53158a. 24064723

[ref24] Murray-Rust P.; Rzepa H. S. Chemical Markup, XML, and the Worldwide Web. 1. Basic Principles. J. Chem. Inf. Comput. Sci. 1999, 39, 928–942. 10.1021/ci990052b.

[ref25] Frisch M. J.; Trucks G. W.; Schlegel H. B.; Scuseria G. E.; Robb M. A.; Cheeseman J. R.; Scalmani G.; Barone V.; Mennucci B.; Petersson G. A.; Nakatsuji H.; Caricato M.; Li X.; Hratchian H. P.; Izmaylov A. F.; Bloino J.; Zheng G.; Sonnenberg J. L.; Hada M.; Ehara M.; Toyota K.; Fukuda R.; Hasegawa J.; Ishida M.; Nakajima T.; Honda Y.; Kitao O.; Nakai H.; Vreven T.; Montgomery J. A. Jr.; Peralta P. E.; Ogliaro F.; Bearpark M.; Heyd J. J.; Brothers E.; Kudin K. N.; Staroverov V. N.; Kobayashi R.; Normand J.; Raghavachari K.; Rendell A.; Burant J. C.; Iyengar S. S.; Tomasi J.; Cossi M.; Rega N.; Millam N. J.; Klene M.; Knox J. E.; Cross J. B.; Bakken V.; Adamo C.; Jaramillo J.; Gomperts R.; Stratmann R. E.; Yazyev O.; Austin A. J.; Cammi R.; Pomelli C.; Ochterski J. W.; Martin R. L.; Morokuma K.; Zakrzewski V. G.; Voth G. A.; Salvador P.; Dannenberg J. J.; Dapprich S.; Daniels A. D.; Farkas Ö.; Ortiz J. V.; Cioslowski J.; Fox D. J. Gaussian 09, revision D.01; Gaussian, Inc.: Wallingford, CT, 2009.

[ref26] O’Boyle N. M.; Banck M.; James C. A.; Morley C.; Vandermeersch T.; Hutchison G. R. Open Babel: An Open chemical toolbox. J. Cheminf. 2011, 3, 33 10.1186/1758-2946-3-33. PMC3198950 21982300

[ref27] Weininger D. SMILES, a Chemical Language and Information System: 1: Introduction to Methodology and Encoding Rules. J. Chem. Inf. Model. 1988, 28, 31–36. 10.1021/ci00057a005.

[ref28] RDKit: Open-source cheminformatics. http://www.rdkit.org (accessed 3 March 2015).

[ref29] Riniker S.; Landrum G. A. Better Informed Distance Geometry: Using What We Know To Improve Conformation Generation. J. Chem. Inf. Model. 2015, 55, 2562–2574. 10.1021/acs.jcim.5b00654. 26575315

[ref30] Rappe A. K.; Casewit C. J.; Colwell K. S.; Goddard W. A.; Skiff W. M. UFF, a full periodic table force field for molecular mechanics and molecular dynamics simulations. J. Am. Chem. Soc. 1992, 114, 10024–10035. 10.1021/ja00051a040.

[ref31] Becke A. D. Density-functional thermochemistry.III. The role of exact exchange. J. Chem. Phys. 1993, 98, 5648 10.1063/1.464913.

[ref32] Lee C.; Yang W.; Parr R. G. Development of the Colle-Salvetti correlation-energy formula into a functional of the electron density. Phys. Rev. B: Condens. Matter Mater. Phys. 1988, 37, 785–789. 10.1103/PhysRevB.37.785. 9944570

[ref33] Vosko S. H.; Wilk L.; Nusair M. Accurate spin-dependent electron liquid correlation energies for local spin density calculations: a critical analysis. Can. J. Phys. 1980, 58, 1200–1211. 10.1139/p80-159.

[ref34] Stephens P. J.; Devlin F. J.; Chabalowski C. F.; Frisch M. J. Ab Initio Calculation of Vibrational Absorption and Circular Dichroism Spectra Using Density Functional Force Fields. J. Phys. Chem. 1994, 98, 11623–11627. 10.1021/j100096a001.

[ref35] Hehre W. J.; Ditchfield R.; Pople J. A. Self—Consistent Molecular Orbital Methods. XII. Further Extensions of Gaussian—Type Basis Sets for Use in Molecular Orbital Studies of Organic Molecules. J. Chem. Phys. 1972, 56, 2257–2261. 10.1063/1.1677527.

[ref36] Rassolov V. A.; Ratner M. A.; Pople J. A.; Redfern P. C.; Curtiss L. A. 6-31G* basis set for third-row atoms. J. Comput. Chem. 2001, 22, 976–984. 10.1002/jcc.1058.

[ref37] Krishnan R.; Binkley J. S.; Seeger R.; Pople J. A. Self-consistent molecular orbital methods. XX. A basis set for correlated wave functions. J. Chem. Phys. 1980, 72, 650 10.1063/1.438955.

[ref38] Valiev M.; Bylaska E. J.; Govind N.; Kowalski K.; Straatsma T. P.; Van Dam H. J. J.; Wang D.; Nieplocha J.; Apra E.; Windus T. L.; De Jong W. A. NWChem: A comprehensive and scalable open-source solution for large scale molecular simulations. Comput. Phys. Commun. 2010, 181, 1477–1489. 10.1016/j.cpc.2010.04.018.

[ref39] Aprà E.; et al. NWChem: Past, present, and future. J. Chem. Phys. 2020, 152, 184102 10.1063/5.0004997. 32414274

[ref40] Hunter C. A. van der Waals interactions in non-polar liquids. Chem. Sci. 2013, 4, 834–848. 10.1039/C2SC21666C.

