# Supplementary material for: SSIPTools: Software and Methodology for Surface Site Interaction Point (SSIP) Approach and Applications
Source: J Chem Inf Model. 2021 Oct 29;61(11):5331–5. doi: 10.1021/acs.jcim.1c01006 (PMC8611717; doi:10.1021/acs.jcim.1c01006)
Supplement: Supplementary file 1 — ci1c01006_si_001.pdf [file ci1c01006_si_001.pdf]

Supplementary Information: Cambridge Mathematics Placements (CMP) Programme

Mark Driver,*[a] Mark J. Williamson[a], Nicola De Mitri[a], Teodor Nikolov[a], and Christopher A. Hunter[a]

[a] *Yusuf Hamied Department of Chemistry, University of Cambridge, Lensfield Road, Cambridge CB2 1EW, UK. Tel: +44 (0)1223 336710; E-mail: *m.d.driver@rug.nl*

Supplementary Information

S1 XML file formats **S1**

S2 SSIP description generation **S1**
S2.1 Molecule Input . . . S1
S2.2 MEPS generation . . . S1
S2.2.1 Molecule Volume . . . S3
S2.3 Footprinting algorithm . . . S4
S2.3.1 Surface Area Calculation . . . S6
S2.3.2 MEPS to hydrogen bond parameter mapping . . . S6
S2.3.3 Efficient searching for nearest neighbours . . . S7
S2.3.4 Functional group identification . . . S7
S2.3.5 Evaluation of trial SSIP configuration . . . S7
S2.3.6 Canonicalisation of output . . . S7

S3 Phasecalculator usage **S7**
S3.1 Input generation and calculations . . . S7
S3.2 SSIP Descriptions . . . S7
S3.3 Phasecalculator XML format specification . . . S8
S3.4 Example calculation: Generating Figure 5 . . . S8

S1 XML file formats

SSIP XML schema files are hosted on the huntergroup website (here: `http://www-hunter.ch.cam.ac.uk/schema/`), and information. The schema are also hosted in a repository in the SSIPTools Gitlab project, HunterDatabaseSchema.

S2 SSIP description generation

S2.1 Molecule Input

The cmlgenerator library provides a simple command line interface to be able to process multiple structure files in a single operation.

S2.2 MEPS generation

NOTE about NWChemCMLUTils library set up and CLI. The MEPS calculation is a multi-step process, which was automated and the functionality was packaged into the nwchemcmlutils repository. The Python application program interface within NWChem [1, 2] was used to improve the efficiency of the workflow.

The Flow Diagram in figure S1 summarises the process of MEPS generation. The MEPS is generated on the 0.002 e bohr^{-3} electron density isosurface [3]. NWChem [1] was used for calculations of the MEPS with a DFT method, the B3LYP functional [4–7]. B3LYP was chosen due to the reliable results produced with a low computational cost [8]. The basis set used was 6-31G* [9, 10] for all atoms, except Bromine, Selenium and Iodine, where a 6-311G** [11] basis set was used.

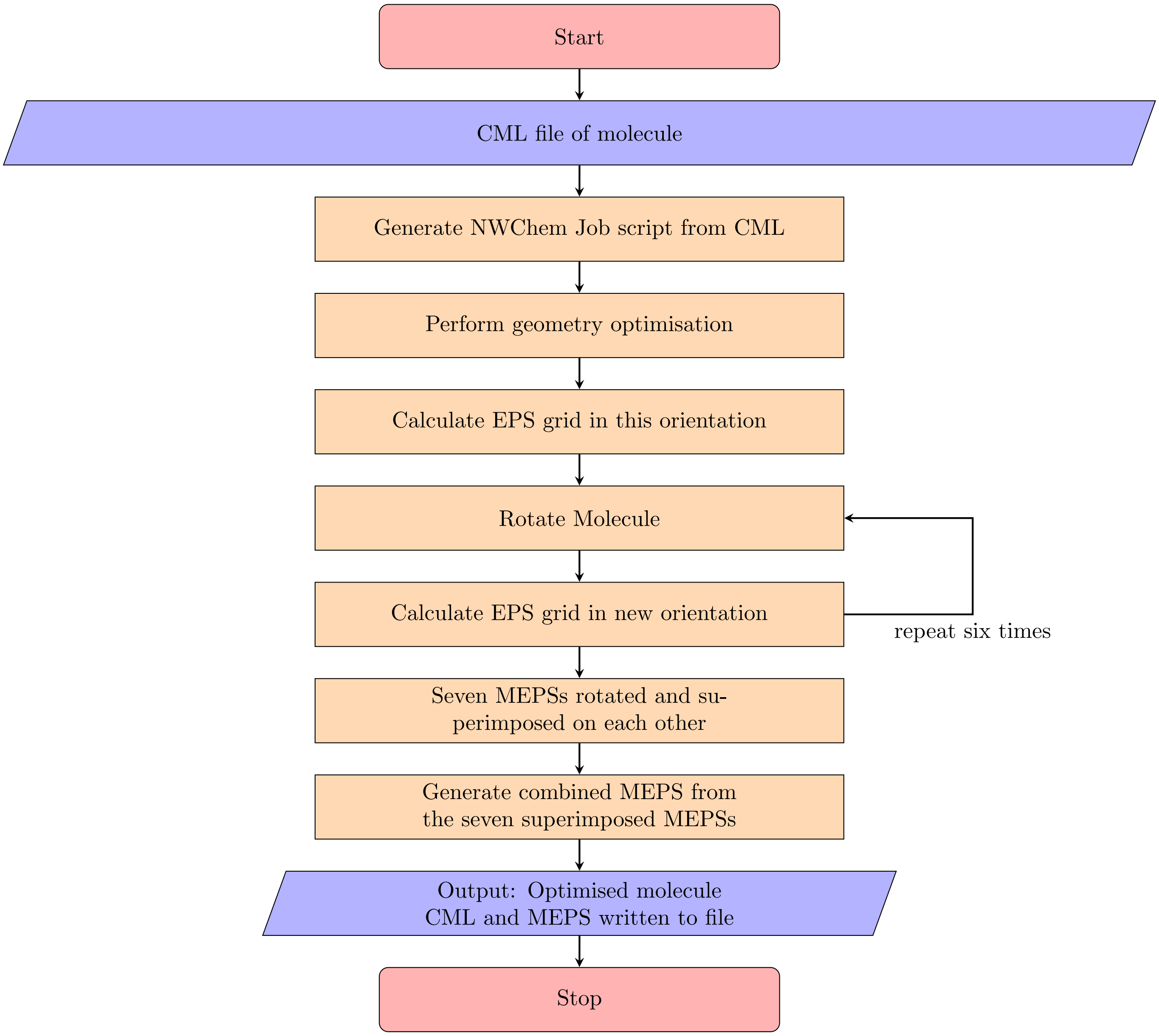

Figure S1: Flow diagram for MEPS generation. Red rounded rectangles are start/stop points, blue trapeziums show input/output operations, orange rectangles are processes.

To store the MEPS data produced during calculations, the unformatted cube file format is used. This is a raw text file that uses FORTRAN formatted information, and is outputted by QM codes to store property information, based on a specification by the Gaussian package [12].

The MEPS is calculated in seven different orientations such that a coarser grid can be used to reduce computational time, without loss of accuracy, and to reduce the likelihood of discretisation errors. The final surface which is outputted is the combination of the seven MEPS grids, which provides a comparable result to the use of a much finer grid. Figure S2 shows the MEPS of water generated from a single orientation, showing the points lie in planes due to the grid. An uneven distribution of points on the surface is heavily reliant on the initial geometry and the grid properties. Rotation by 45 or 120 degrees about the x, y and z axes is used to generate a further six surfaces. The resultant surface from the superposition of these seven surfaces after reorientation shown in figure S3, contains an even distribution of points over the entire surface, with a higher density.

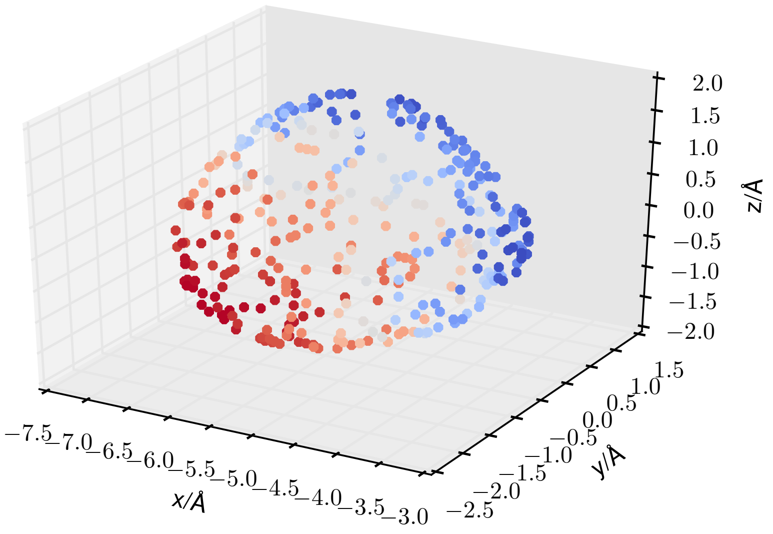

Figure S2: MEPS for water in one orientation (atoms not shown). Calculation used B3LYP with a 6-31G* basis set, a grid padding of 2.0 Å and step size of 0.088 Å, on the 0.002 e bohr^{-3} electron isosurface, with a tolerance of 0.00003 e bohr^{-3} in the electron density. The colour map goes from red (negative potentials) to blue (positive potentials).

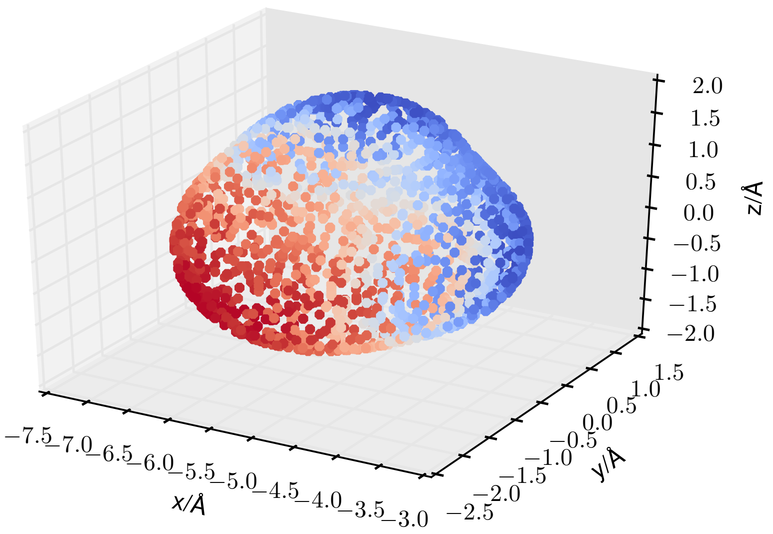

Figure S3: MEPS for water after all seven orientations have been superposed (atoms not shown). Calculation used B3LYP with a 6-31G* basis set, a grid padding of 2.0 Å and step size of 0.088 Å, on the 0.002 e bohr^{-3} electron isosurface, with a tolerance of 0.00003 e bohr^{-3} in the electron density. The colour map goes from red (negative potentials) to blue (positive potentials).

S2.2.1 Molecule Volume

The volume enclosed by an isosurface is also calculated during this stage within NWChem. This information is required for the calculation of the temperature dependent SSIMPLE model previously described [13]. The number of voxels with electron densities greater than the specified surface is calculated for each orientation.

The mean volume enclosed is then written in the merged cube file that is outputted from the calculation.

S2.3 Footprinting algorithm

Water is known to have four hydrogen bond interaction sites. Calero *et al.* [3] used this to define the surface area of a SSIP as a quarter the surface area of water which corresponds to $A_{SSIP} = 9.35$ Å^2.

Hydrogen bond interactions occlude a segment of the surface from other interactions. This places a restriction on the proximity of SSIPs on a molecular surface, to avoid clashing. The minimum separation of SSIPs on the surface, d (in figure S4), influences the SSIP description produced. It was parameterised by Calero *et al.* [3], and assigned a value of 3.2 Å.

The flow diagram in figure S4 summarises the footprinting process, previously described [3].

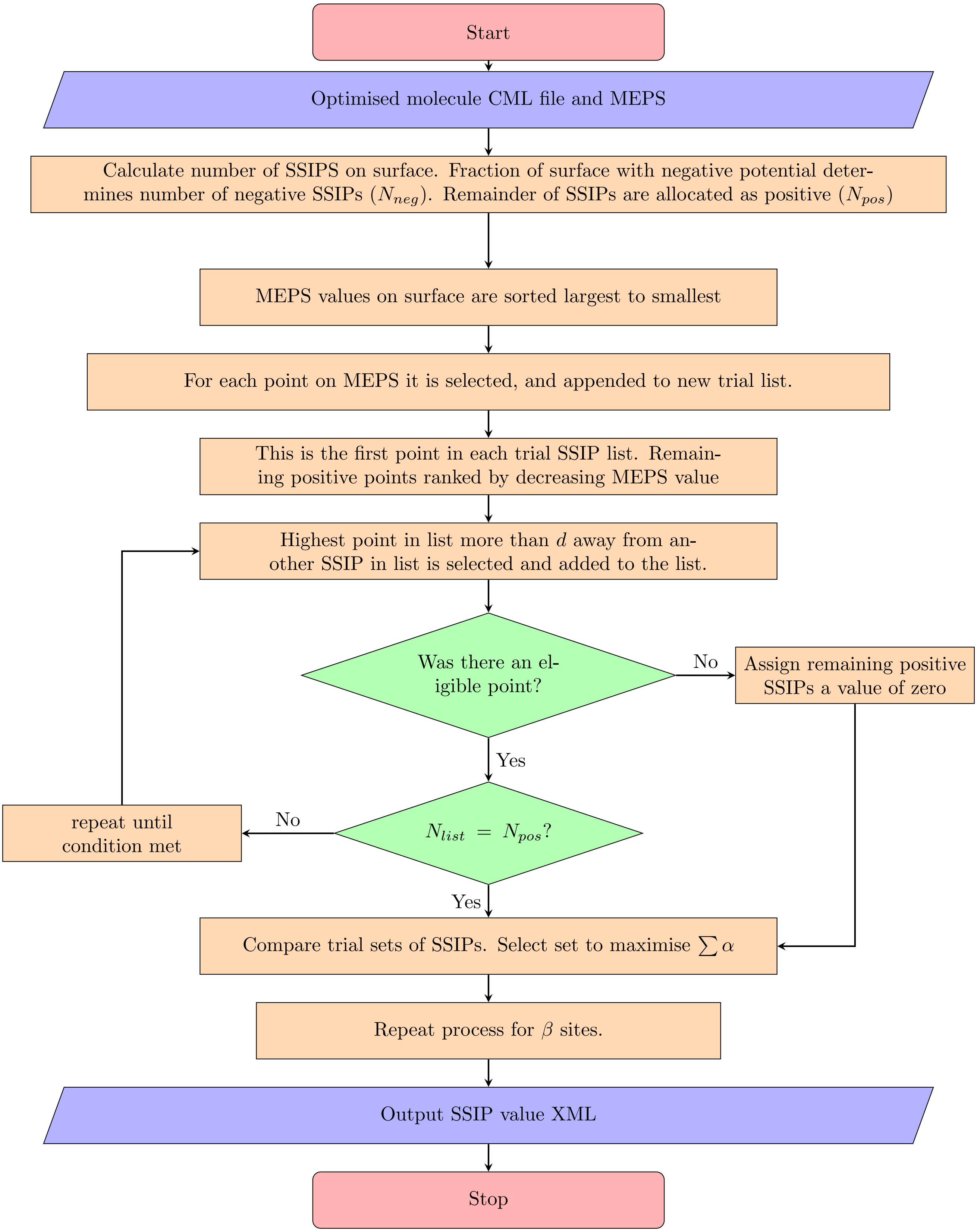

Figure S4: Flow diagram summarising footprinting process for a single molecule. Red rounded rectangles are start/stop points, blue trapeziums show input/output operations, orange rectangles are processes and green diamonds are decision nodes.

A SSIP represents a single surface segment. The partitioning of a molecule into a set of SSIPs requires knowledge of the number of SSIPs to be assigned to the molecule. This is calculated from the molecular surface area.

S2.3.1 Surface Area Calculation

The molecular surface area is calculated to be able to determine the required number of SSIPs to assign to a molecule in the first step of footprinting. This is done using the positional information for the MEPS points, which lie on a convex hull that encapsulates the molecule. The molecular surface area, S.A., is calculated using equation (S1).

$$S.A. = \sum_{r_i}^{N} D_{r_i} \pi r_D^2 \tag{S1}$$

Where r_i is the position of the ith point on the surface, D_{r_i} is the density of points in the local environment, given by equation (S2). The local environment is defined by r_D, the density radius, and $\delta(r_i, r_j)$ (equation (S2) and equation (S3)).

$$D_{r_i} = \frac{1}{\sum_j^N \delta(r_i, r_j)} \tag{S2}$$

$$\delta(i,j) = \begin{cases} 1 \text{ if } |r_i - r_j| \leq r_D \\ 0 \text{ otherwise} \end{cases} \tag{S3}$$

The density of points in the local environment is simply the reciprocal of the sum of the number of other points within the defined search radius. Each point on the surface contributes a circular area, equal to the area occluded by the density radius, weighted by the density of points in the local environment. A value of $r_D = 0.5$ Å was used.

S2.3.2 MEPS to hydrogen bond parameter mapping

The mapping of molecular electrostatic potential surface (MEPS) value to hydrogen bond parameters uses second order polynomial functions, parameterised in [3]. SSIP values for positive MEPS segments are mapped to hydrogen bond donor parameters with equation (S4). The negative MEPS values are mapped to hydrogen bond acceptor parameters with equation (S5).

$$\epsilon = 1.12 \times 10^{-5} \psi_{0.002}^2 + 1.14^{-2} \psi_{0.002} \text{if } \psi_{0.002} > 0.0 \tag{S4}$$

$$\epsilon = c \left(8.33 \times 10^{-5} \psi_{0.002}^2 - 2.08 \times 10^{-2} \psi_{0.002}\right) \text{ if } \psi_{0.002} < 0.0 \tag{S5}$$

Where $\psi_{0.002}$ is the MEPS value on the 0.002 e bohr^{-3} electron density isosurface in kJmol^{-1}, and c is an empirical correction factor based on functional group. The correction factors are contained in table S1.

Functional Group	c
Nitrile nitrogen	0.77
Primary amine nitrogen	1.02
Secondary amine nitrogen	1.24
Tertiary amine nitrogen	1.34
Pyridine type nitrogen	1.07
Ether type oxygen	1.16
Alcohol type oxygen	0.86
Aldehyde carbonyl oxygen	0.89
Ester carbonyl oxygen	0.92
Carbonate carbonyl oxygen	0.90
Nitro oxygen	0.77
Any oxygen atom bonded to sulfur	1.00
Any oxygen atom bonded to phosphorus	1.10

Table S1: Empirical functional group correction factors for negative surface segments in equation (S5).

S2.3.3 Efficient searching for nearest neighbours

Calculating the distance between all points on the MEPS is an $\mathcal{O}\left(N^2\right)$ operation, which could become very expensive for large molecules. To reduce the computational cost, a KDTree is used [14, 15], to reduce the scaling to $\mathcal{O}\left(NlogN\right)$ making the search for points satisfying the distance cutoffs faster. The KDTree produces a space-partitioned data structure. A KDTree data structure can be more efficiently traversed than a standard array structure, which was used previously.

S2.3.4 Functional group identification

As noted in [3] the acceptor sites have a correction factor applied. The requirement of an empirical correction factor to improve the prediction of hydrogen bond acceptors produces an extra requirement of functional group identification within the code.

This is done by expressing the molecule in a graph representation, with atoms as vertices, and bonds as edges. A subgraph isomorphism approach can then be used to identify any occurrences of the functional group, if it is also described as a graph. The algorithm used is the VF2 of Cordella [16], which has been implemented in this code by Teodor Nikolov.

The functional group identification routine employed prevents the assignment of multiple correction factors. This is done by the canonical ordering of functional group subgraphs by use of an enumeration object. Once an assignment to a functional group is made, no others can be assigned. The functional groups are sorted by IUPAC precedence [17], so are therefore ordered so the largest collection of atoms would be assigned first, i.e. an oxygen atom would be assigned to an ester rather than an ether.

S2.3.5 Evaluation of trial SSIP configuration

The positional assignment uses the MEPS value, rather than the ϵ value of the SSIP. This is done to ensure the correct surface features are found. Once the best configuration is found, the region around each SSIP assigned is then further examined. Since a SSIP interaction involves a small contact area and not just a single point, all MEPS points within this contact area must be inspected. This led to the assignment of a contact radius, r, assigned value of 1.1Å, in work by Calero *et al.* [3]. A SSIP assigned to the surface is replaced by a more polar point that is found within this radius, to more accurately account for the polarity of the region.

S2.3.6 Canonicalisation of output

To ensure reproducibility of footprints from the calculation, the output ordering of SSIPs when written to file must be consistent, to allow for unit testing. The SSIPs are ordered by decreasing numerical value. If there are any SSIPs with the same value, the distance of the SSIPs to the molecule (mass unweighted) centroid is then used to ensure consistent ordering.

This is required because the traversal of the MEPS surfaces generating the different trial SSIP collections is not deterministic, so the same solution may be found with different additions to the SSIP description.

S3 Phasecalculator usage

Phasecalculator provides a convenient wrapper for the functionality required to calculate FGIPs and SSIs for solvent systems.

S3.1 Input generation and calculations

Operation of phasecalculator via the CLI is split into two categories by sub-commands; *calculate* and *inpgen*. The *calculate* option will run the calculations defined in the input phasecalculator XML. This requires access to a version of the ssip-phastransfer jar file on the local system. The *inpgen* command can generate the required XML for calculations based on predefined SSIP descriptions.

S3.2 SSIP Descriptions

The SSIP descriptions for solvent molecules studied were previously defined in [13, 18]. The descriptions are present in the puresolventinfomration repository. Both sets of SSIP descriptions are accessible with phasecalculator when constructing input with the inpgen sub-command. The default description for inpgen, uses the polarised alcohol descriptions from [18, 19]. The unpolarised alcohol descriptions used in [13] can be selected using the *–unpolarised* flag when using the inpgen CLI.

S3.3 Phasecalculator XML format specification

The XML file input to the calculate sub-command of phasecalculator provides an agnostic interface to calculate properties for non-standard solvents.

For solvents with components not defined, or to use descriptions not defined in the puresolventinformation repository, *inpgen* cannot be used.

To use non-standard SSIP descriptions, defined in the previous section, for solvents previously studied requires the creation of valid XML input for the calculate sub-command with the included paths to include in listing 1.

Listing 1: Example phase definition from an phasecalculator input XML file. The SSIP description to use for a molecule is defined by the *ssipFilelocation* attribute. If a different SSIP description for a moelcule predefined is required, this path requires modification to the file path of the SSIP description of interest.

```xml
<phasecalc:Phase xmlns:phasecalc="http://www-hunter.ch.cam.ac.uk/PhaseCalculatorSchema">
<phasecalc:Molecule phasecalc:name="ethanol"
    phasecalc:inChIKey="LFQSCWFLJHTTHZ-UHFFFAOYSA-N"
    phasecalc:ssipFileLocation="/home/usr/anaconda3/envs/resultsanalysis/lib/python3.7/
site-packages/puresolventinformation/resources/
unpolarisedssipfiles/LFQSCWFLJHTTHZ-UHFFFAOYSA-N_ssip.xml"
    phasecalc:molefraction="1.0000"/>
<phasecalc:Temperature phasecalc:value="250.000" phasecalc:unit="KELVIN"/>
</phasecalc:Phase>
```

S3.4 Example calculation: Generating Figure 5

The generation of Figure 5 of the paper can be done easily in two steps, after all packages are installed. This process is described in more detail in the README file of phasecalculator. The commands required are as follows:

```
python -m phasecalculator inpgen -f -j $PATH_TO_JAR -p \
$CONDA_PREFIX/lib/python3.7/site-packages/phasecalculator/test/resources/examplephasecomp.csv
python -m phasecalculator calculate -f systemcollection.xml
```

Where PATH_TO_JAR is the location of the compiled ssip-phasecalculator JAR to use, and the csv file is included in the installation of phasecalculator. Details of this example are included in the help messages of the phasecalculator package, in addition to describing complete usage options.

References

(1) Valiev, M.; Bylaska, E. J.; Govind, N.; Kowalski, K.; Straatsma, T. P.; Van Dam, H. J. J.; Wang, D.; Nieplocha, J.; Apra, E.; Windus, T. L.; De Jong, W. A. *Comput. Phys. Commun.* **2010**, *181*, 1477–1489.

(2) Aprà, E.; Bylaska, E. J.; de Jong, W. A.; Govind, N.; Kowalski, K.; Straatsma, T. P.; Valiev, M.; van Dam, H. J. J.; Alexeev, Y.; Anchell, J.; Anisimov, V.; Aquino, F. W.; Atta-Fynn, R.; Autschbach, J.; Bauman, N. P.; Becca, J. C.; Bernholdt, D. E.; Bhaskaran-Nair, K.; Bogatko, S.; Borowski, P.; Boschen, J.; Brabec, J.; Bruner, A.; Cauët, E.; Chen, Y.; Chuev, G. N.; Cramer, C. J.; Daily, J.; Deegan, M. J. O.; Dunning, T. H.; Dupuis, M.; Dyall, K. G.; Fann, G. I.; Fischer, S. A.; Fonari, A.; Früchtl, H.; Gagliardi, L.; Garza, J.; Gawande, N.; Ghosh, S.; Glaesemann, K.; Götz, A. W.; Hammond, J.; Helms, V.; Hermes, E. D.; Hirao, K.; Hirata, S.; Jacquelin, M.; Jensen, L.; Johnson, B. G.; Jónsson, H.; Kendall, R. A.; Klemm, M.; Kobayashi, R.; Konkov, V.; Krishnamoorthy, S.; Krishnan, M.; Lin, Z.; Lins, R. D.; Littlefield, R. J.; Logsdail, A. J.; Lopata, K.; Ma, W.; Marenich, A. V.; Martin del Campo, J.; Mejia-Rodriguez, D.; Moore, J. E.; Mullin, J. M.; Nakajima, T.; Nascimento, D. R.; Nichols, J. A.; Nichols, P. J.; Nieplocha, J.; Otero-de-la-Roza, A.; Palmer, B.; Panyala, A.; Pirojsirikul, T.; Peng, B.; Peverati, R.; Pittner, J.; Pollack, L.; Richard, R. M.; Sadayappan, P.; Schatz, G. C.; Shelton, W. A.; Silverstein, D. W.; Smith, D. M. A.; Soares, T. A.; Song, D.; Swart, M.; Taylor, H. L.; Thomas, G. S.; Tipparaju, V.; Truhlar, D. G.; Tsemekhman, K.; Van Voorhis, T.; Vázquez-Mayagoitia, Á.; Verma, P.; Villa, O.; Vishnu, A.; Vogiatzis, K. D.; Wang, D.; Weare, J. H.; Williamson, M. J.; Windus, T. L.; Woliński, K.; Wong, A. T.; Wu, Q.; Yang, C.; Yu, Q.; Zacharias, M.; Zhang, Z.; Zhao, Y.; Harrison, R. J. *J. Chem. Phys.* **2020**, *152*, 184102.

(3) Calero, C. S.; Farwer, J.; Gardiner, E. J.; Hunter, C. A.; Mackey, M.; Scuderi, S.; Thompson, S.; Vinter, J. G. *Phys. Chem. Chem. Phys.* **2013**, *15*, 18262–73.

(4) Becke, A. D. *J. Chem. Phys.* **1993**, *98*, 5648.

(5) Lee, C.; Yang, W.; Parr, R. G. *Phys. Rev. B* **1988**, *37*, 785–789.

(6) Vosko, S. H.; Wilk, L.; Nusair, M. *Can. J. Phys.* **1980**, *58*, 1200–1211.

(7) Devlin, P. J. S.; Chabalowski, F. J. C. F.; Frisch, M. J. *J. Phys. Chem.* **1994**, *98*, 11623–11627.

(8) Simón, L.; Goodman, J. M. *Org. Biomol. Chem.* **2011**, *9*, 689–700.

(9) Hehre, W. J.; Ditchfield, R.; Pople, J. A. *J. Chem. Phys.* **1972**, *56*, 2257–2261.

(10) Rassolov, V. A. *J. Comput. Chem.* **2001**, *22*, 976–984.

(11) Krishnan, R.; Binkley, J. S.; Seeger, R.; Pople, J. A. *J. Chem. Phys.* **1980**, *72*, 650.

(12) Frisch, M. J.; Trucks, G. W.; Schlegel, H. B.; Scuseria, G. E.; Robb, M. A.; Cheeseman, J. R.; Scalmani, G.; Barone, V.; Mennucci, B.; Petersson, G. A.; Nakatsuji, H.; Caricato, M.; Li, X.; Hratchian, H. P.; Izmaylov, A. F.; Bloino, J.; Zheng, G.; Sonnenberg, J. L.; Hada, M.; Ehara, M.; Toyota, K.; Fukuda, R.; Hasegawa, J.; Ishida, M.; Nakajima, T.; Honda, Y.; Kitao, O.; Nakai, H.; Vreven, T.; Montgomery Jr., J. A.; Peralta, J. E.; Ogliaro, F.; Bearpark, M.; Heyd, J. J.; Brothers, E.; Kudin, K. N.; Staroverov, V. N.; Kobayashi, R.; Normand, J.; Raghavachari, K.; Rendell, A.; Burant, J. C.; Iyengar, S. S.; Tomasi, J.; Cossi, M.; Rega, N.; Millam, J. M.; Klene, M.; Knox, J. E.; Cross, J. B.; Bakken, V.; Adamo, C.; Jaramillo, J.; Gomperts, R.; Stratmann, R. E.; Yazyev, O.; Austin, A. J.; Cammi, R.; Pomelli, C.; Ochterski, J. W.; Martin, R. L.; Morokuma, K.; Zakrzewski, V. G.; Voth, G. A.; Salvador, P.; Dannenberg, J. J.; Dapprich, S.; Daniels, A. D.; Farkas, Ö.; Foresman, J. B.; Ortiz, J. V.; Cioslowski, J.; Fox, D. J. Gaussian09 Revision D.01, Gaussian Inc. Wallingford CT 2009.

(13) Driver, M. D.; Hunter, C. A. *in preparation* **2020**, -.

(14) Wald, I.; Havran, V. In *2006 IEEE Symposium on Interactive Ray Tracing*, IEEE: 2006, pp 61–69.

(15) De Berg M.; Cheong, O.; van Kreveld, M.; Overmars, M. In *Computational Geometry: Algorithms and Applications*; Springer Berlin Heidelberg: Berlin, Heidelberg, 2008, pp 95–120.

(16) Cordella, L. P.; Foggia, P.; Sansone, C.; Vento, M. *IEEE Transactions on Pattern Analysis and Machine Intelligence* **2004**, *26*, 1367–1372.

(17) Leigh, G.; Favre, H.; Metanomski, W., *Principles of Chemical Nomenclature A GUIDE TO IUPAC RECOMMENDATIONS*; Blackweil Science Ltd: 1998.

(18) Driver, M. D.; Williamson, M. J.; Cook, J. L.; Hunter, C. A. *Chem. Sci.* **2020**, *11*, 4456–4466.

(19) Driver, M. D.; Hunter, C. A. *Phys. Chem. Chem. Phys.* **2020**, *22*, 11967–11975.
